# Discovery of a Novel Species Infecting Goats: Morphological and Molecular Characterization of *Babesia aktasi* n. sp.

**DOI:** 10.3390/pathogens12010113

**Published:** 2023-01-10

**Authors:** Sezayi Ozubek, Mehmet Can Ulucesme, Munir Aktas

**Affiliations:** Department of Parasitology, Faculty of Veterinary Medicine, University of Firat, Elazig 23200, Turkey

**Keywords:** *Babesia*, goat, morphology, phylogenetic analysis, sequence comparison

## Abstract

A novel *Babesia* sp. infecting goats was discovered based on the molecular findings obtained in the current study, which was conducted in the Mediterranean region of Türkiye. The goal of this study was to isolate this species of *Babesia* (*Babesia* sp.) infecting goats in vivo and to assess the genetic and morphological characterization of the parasite. To identify the animal naturally infected with *Babesia* sp. and isolate the parasite from this animal, field studies were conducted first, and genomic DNA were extracted from blood samples taken from goats (*n* = 50). The *Theileria*, *Babesia*, and *Anaplasma* species were identified using a nested PCR-based reverse line blotting (RLB) method. The study included one goat that was determined to be infected with *Babesia* sp. (single infection) in RLB for in vivo isolation. A blood smear was prepared to examine the parasite’s morphology, but it was found to be negative microscopically. Following that, a splenectomy operation (to suppress the immune system) was performed to make the parasites visible microscopically in this animal. Parasitemia began after splenectomy, and the maximum parasitemia was determined to be 1.9%. The goat displayed no significant symptoms other than fever, loss of appetite, and depression. During a period when parasitemia was high, blood from this goat was inoculated into another splenectomized goat (*Theileria*-*Babesia*-*Anaplasma-Mycoplasma* spp. free). On the third day of inoculation, 10% parasitemia with high fever was detected in the goat, and on the fourth day, the goat was humanely euthanized due to severe acute babesiosis symptoms. Except for mild subcutaneous jaundice, no lesions were discovered during the necropsy. According to the microscopic measurement results, ring, double pyriform, spectacle-frame-like, and line forms were observed, and it was observed to be between 1.0–2.5 µm (1.38 ± 0.17 to 0.7 ± 0.21-all forms). A phylogenetic analysis and sequence comparison using the *18S rRNA* and *cox1* genes revealed that this species is distinct from the small ruminant *Babesia* species (*18S rRNA* 92–94%, *cox1* 79–80%) and has the highest similarity to *Babesia* sp. deer, which has been reported in deer. Furthermore, it was determined to resemble *B. venatorum*, *B. divergens*, *Babesia* sp. FR1 and *Babesia* sp. MO1 species, all of which are zoonotic. Additional research is needed to clarify the clinical status of this parasite in goats and other hosts (mountain goat, sheep, calf).

## 1. Introduction

Shortly after the discovery of *Babesia* in bovine erythrocytes by Victor Babes in 1888, the same researcher reported that a parasite with similar characteristics also infected sheep [1]. Since then, it has been reported that *Babesia ovis*, *B. motasi*, *B. crassa*, *B. taylori* and *B. foliate* cause babesiosis in sheep and goats [2]. Babesiosis is a tick-borne disease caused by the genus *Babesia*, which is frequently observed in domestic and wild animals in tropical and subtropical regions [2,3,4]. The most pathogenic species is *Babesia ovis*, which is endemic to southern Europe, Africa, the Middle East, and Asia [5], and causes severe economic losses in sheep and goats [5]. *Babesia crassa*, which has low pathogenicity, was isolated in Iran, and *B. motasi*, which includes more than one species and subspecies, was isolated in China and Europe. *B. motasi* infections can cause mild clinical signs in sheep but can lead to severe anemia and death in goats [6].Human babesiosis caused by *B. motasi* and *B. crassa* has also been reported sporadically in Asia [7,8]. There is very little information on *B. taylori* and *B. foliate*, which have been reported to infect sheep and goats but for which no molecular data is available [4].

In the last two decades, the use of molecular diagnostic techniques to investigate ticks and tick-borne agents has increased dramatically, and as a result, new species of piroplasm have been discovered [9,10,11,12,13,14]. Using PCR-based reverse line blot (RLB) hybridization, which is used for the detection and identification of piroplasm species, novel *Theileria* and *Babesia* species were identified. [15,16]. This study’s objective was to perform morphological and molecular characterization of a new species of *Babesia* previously reported in goats based on molecular data [16].

## 2. Materials and Methods

### 2.1. Study Area and Animal Samples

In 2016, a new *Babesia* sp. was detected molecularly in goats in Anamur, Mersin province (36°01′17″ N, 32°48′07″ E), in the Mediterranean region of Turkiye [16]. In order to investigate more detailed information (morphological and molecular data) about this parasite, blood samples were collected from goats by visiting Mersin province between 2018 and 2020. Mersin has a typical subtropical Mediterranean climate with hot and humid summers and mild and rainy winters. The Taurus Mountains, a mountain complex in southern Türkiye that separates the Mediterranean coastal region from the Central Anatolian Plateau, are located in this region. Sheep and goats are kept in the sheepfold during lambing and at the beginning of milking (January–March), and on pasture during the remainder of the milking season (April–May) in the Taurus Mountains. Additionally, mountain goats inhabit this region [17].

### 2.2. Determination of New Babesia sp. Infected Goat in Field Samples

Blood samples were taken from the vena jugularis of 50 randomly selected goats (apparently healthy) in Mersin province (Anamur, Bozyazi) and placed into EDTA tubes. Animals whose blood samples were taken, were treated with acaricide (Flugon^®^ 1%, Vetas, Turkiye) and kept in a tick-free environment until the completion of the PCR results. The genomic DNA was extracted from 200 µL of EDTA anticoagulated blood samples from the goats using a kit (PureLink^TM^ Genomic DNA Mini Kit, Invitrogen Corporation, Carlsbad, CA, USA) according to the manufacturer’s instructions. For the determination of new *Babesia* sp., and mixing infection with blood parasites in field samples, a nested PCR was performed to use in an RLB assay for the *Anaplasma*/*Ehrlichia* and *Theileria*/*Babesia* species, using Ec9/Ec12A [18]-16S8FE/B-GA1B [19] and Nbab1F/Nbab1R [9]-RLBF2/RLBR2 [20] primers, respectively. The nested PCR products were used in reverse line blotting (RLB) to detect the *Anaplasma*/*Ehrlichia* and *Theileria*/*Babesia* species. Additionally, positive RLB samples were analyzed for hemotropic mycoplasma using nested PCR with 8F/1492R- F2/R2 primers [21,22].

To obtain the near full sequence of the *18S rRNA* gene region, PCR was performed using Nbab1F-Nbab1R primers [9] to confirm the determination of new *Babesia* sp. and to gain further molecular data analysis, and modified nested PCR protocols amplifying *cytochrome c oxidase subunit 1* (*cox1*) gene were performed using the BaFor1/BaRev1 and BaFor2/BaRev2 primers [23]. PCR products were electrophoresed on an agarose gel containing 1.4% agarose, stained with ethidium-bromide, and sequenced by a private company. (BM-Labosis, Turkiye). The primers and probes used in the study are listed in Supplementary Table S1.

### 2.3. Experimental Study and Monitoring Animals

**Figure 1:**
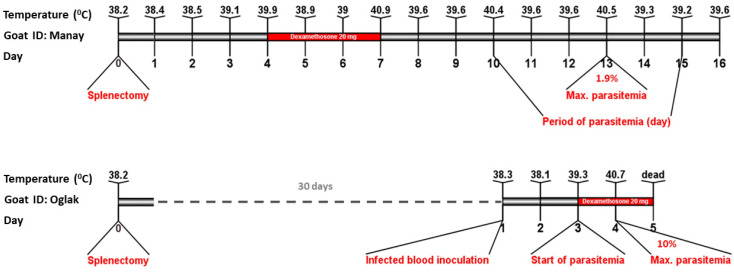
Experimental design for the new *Babesia* sp. isolated from goats and schematic representation of animals (*Manay* and *Oglak*) body temperature, parasitemia period and maximum parasitemia. Diagrams were constructed using IBS program [30], version 1.0.

The goat (ID: *Manay*, 3 year-old female) infected with new *Babesia* sp., as determined by nested PCR-based RLB and sequence analysis, and another goat (ID: *Oglak*, 5 month-old male) free of blood parasites, were both brought to the Firat University Veterinary Faculty for experimental research. The animals (*Manay* and *Oglak*) were relocated to a separate compartment, and their care and feeding were continued throughout duration of the experiment. Throughout the experimental study, flumethrine 1% (Flugon^®^ 1%, Vetas) was applied every 21 days to prevent tick infestations. Before the splenectomy, the goat was PCR and RLB tested for *Babesia*-*Theileria*-*Anaplasma*-*Mycoplasma* spp. Firstly, a splenectomy was performed to suppress the immune system of the goat named *oglak* and it was examined for blood parasites using the PCR method at certain intervals for about 30 days until the time of the experimental infection [24]. A splenectomy was conducted on the goat, named *manay*, who tested positive for *Babesia* sp., and 20 mg of dexamethasone (Vetakort^®^ 4 mg, Vetas-intramuscular injection) was administered for three days after the operation. The surgical procedures utilized in the splenectomy surgery were carried out exactly as described by Sevinc et al. [25]. Manay was evaluated daily after splenectomy for clinical responses, rectal temperature, and the presence of piroplasm parasites in peripheral blood smears. During the peak of parasitemia, 20 mL of infected blood from this animal was administered to Oglak. Similar to Manay, Oglak was inspected daily, and its parasitemia was determined. In addition, when parasitemia was detected, 20 mg of dexamethasone was administered to Oglak (Figure 1). To measure parasitemia in both animals, blood smears were taken from the animals’ ear tips and stained with Giemsa dye, and parasitemia was estimated using the method published by Luo et al. [26]. After the parasites appeared, as suggested by Uilenberg et al. [27] and Guan et al. [28], measurements were taken with an Olympus microscope BX43 (Olympus, Tokyo, Japan) and photographs were taken with an Olympus DP72 Digital Camera System (Olympus, Tokyo, Japan).

### 2.4. Phylogenetic and Percent Identity Matrix Analyses

Phylogenetic analyzes of new *Babesia* sp. isolated in this study were carried out using sequences of *18S rRNA* and *cox1* genes from *Babesia* species isolated in vertebrate hosts. Two separate phylogenetic trees were constructed for *18S rRNA* and *cox1* sequences using MEGAX software [29]. The nearly full-length complete sequence of the *18S rRNA* (1709 base pairs) and *cox1* (~900 bp) genes determined for new *Babesia* sp. was compared to other targeted *Babesia* species using Percent Identity Matrix analysis (http://www.ebi.ac.uk/Tools/msa/clustalo accessed on 6 November 2022).

### 2.5. Ethics Statement

This study was carried out according to the regulations of animal and welfare issued by the Turkish legislation for the protection of animals (Animal Experiment Ethic Committee, protocol no: 2018/100).

## 3. Results

### 3.1. Prevalance Rate of Babesia sp. in Field Samples

Fifty blood samples collected from goats were screened for the presence of hemoparasites (*Babesia* spp., *Theileria* spp., *Anaplasma* spp.) and hemotropic mycoplasmas (*Mycoplasma* spp.) by molecular tools (PCR and RLB). The frequency of each tick-borne hemoparasite and hemotropic mycoplasma (single and mixed infections) detected is shown in Table 1. The findings showed positivity in 14 (28%) of the sampled goats and revealed the presence of five pathogens. Of the pathogens detected, new *Babesia* sp. was the most prevalent (12/50, 24%), followed by *T. ovis* (9/50, 18%) and *Mycoplasma* spp. (8/50, 16%).

### 3.2. The Host’s Ability to Control Parasitemia Is Diminished by Splenectomy and Pharmacological Immunosuppression with Dexamethasone

The first piroplasm forms of the novel *Babesia* sp. were seen microscopically in *Manay* on the tenth day after the splenectomy. Parasitemia reached its peak (1.9%) on the 13th postoperative day, then declined for two more days (14 and 15 days), with no agent observed in the peripheral blood on the 16th. The animal’s body temperature was found to be fluctuating. On the seventh postoperative day, there was an increase in body temperature (40.9 °C), followed by a slight decrease for the next two days, and then another increase (40.5 °C) on the tenth day. Fever, anorexia, and depression were observed after splenectomy until the parasitemia disappeared. On the day that the parasite percentage in the peripheral blood reached 1.9%, 20 mL of blood was drawn from *manay* and inoculated into *Oglak*. Before inoculation, *Oglak* was tested for *Babesia*, *Theileria*, *Anaplasma*, and *Mycoplasma* spp. using PCR-RLB and found to be negative. When the parasite was noticed in blood smears, *Oglak* was given 20 mg of dexamethasone intramuscularly every day during 4 consecutive days. Piroplasm forms were observed in the *Oglak*’s peripheral blood on the second day after parasite inoculation, parasitemia (10%) and an increase in body temperature (40.7 °C) were observed on the third day (Figure 1), and the goat was humanely euthanized on the fourth day due to severe acute babesiosis symptoms (fever, low PCV, and anemia). Except for minor icterus under the skin, the necropsy revealed no macroscopic babesiosis signs.

Parasites in infected erythrocytes have been described in various morphological forms including ring, paired pyriform, spectacle frame-like, and line. Except for the ring forms, it was observed that other forms had no transparent and clear cytoplasm.

**Figure 2:**
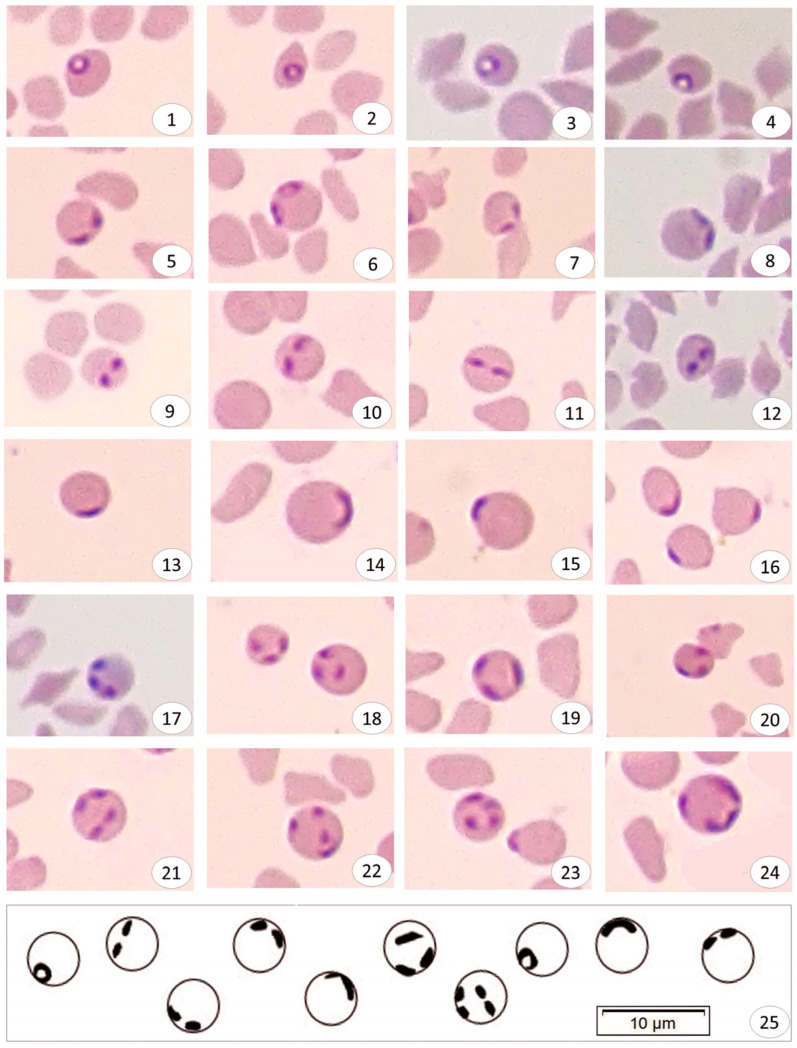
Microscopic visualization of the defined ring (**1**–**4**), paired pyriform (**5**–**8**), spectacle frame-like (**9**–**12**), and line forms (**13**–**16**) in erythrocytes infected with *Babesia* sp. Triple and quadruple forms (**17**–**24**) formed by the combination of various forms belonging to *Babesia* sp., and graphic representation of these forms (**25**). Giemsa’s stain. Bar = 10 µm.

In the ring form, the cytoplasm was transparent and highly prominent, the nucleus was stained reddish-dark purple and located close to the red blood cell membrane (Figure 2, plates 1–4). The size of the piroplasms varied from 0.74 to 1.87 µm with mean dimensions of 1.27 ± 0.27 µm.

For the paired pyriform, it was observed that the paired pyriform, unlike the ring forms, did not have a significant cytoplasm. These forms did not appear to be in contact with the two merozoites (Figure 2, plates 5–8), unlike the double pear forms typically found in many other *Babesia* species. It was also observed that most of the infected erythrocytes contained only one pair of parasites (Figure 2, plates 5–8), although sometimes it was more than one (Figure 2, plate 21). The size of the piroplasms varied from 0.96 to 1.64 × 0.48 to 1.03 µm, with mean dimensions of 1.26 (±0.18) µm × 0.7 (±0.15) µm.

With regard to the spectacle frame-like type, this form appears microscopically as if they were surrounding the red blood cell like the diameter of a circle. It is called spectacle frame-like because it resembles the shape of a spectacle frame (Figure 2, plates 9–12). In most of the erythrocytes, it was observed that the paired pyriform, which is oval (Figure 2, plates 9, 10, 12) or has a thick line (Figure 2, plate 11), is not connected to each other and the angle between them reaches 180 °C. The size of the piroplasms varied from 0.74 to 1.52 × 0.52 to 0.94 µm with mean dimensions of 1.18 (±0.2) µm × 0.67 (±0.12) µm.

With regard to the line form, this form is found close to the red blood cell membrane as a thick arc-shaped line. (Figure 2, plates 13–16). The size of the piroplasms varied from 0.95 to 2.12 × 0.28 to 0.68 µm with mean dimensions of 1.64 (±0.31) µm × 0.51 (±0.11) µm.

In addition, it is rarely observed that forms such as paired pyriform + spectacle frame (Figure 2, panel 21), paired pyriform + line (Figure 2, panel 24), line + ring (Figure 2, panel 20) come together (Figure 2, plate 17–24).

The new type of *Babesia* sp. (1.38 ± 0.17 to 0.7 ± 0.21-all forms) is defined as small *Babesia* because it is located between 1.0–2.5 μm according to the microscopic measurement results. In slides stained with Giemsa, the cytoplasm is not clearly visible in other forms, except for the ring form. Up to four merozoites were seen in an infected erythrocyte (Figure 2, plates 21–24). Furthermore, the different forms of the new *Babesia* sp. were presented graphically (Figure 2, plates 25).

### 3.3. Sequence Comparisons and Phylogenetic Analysis

**Figure 3:**
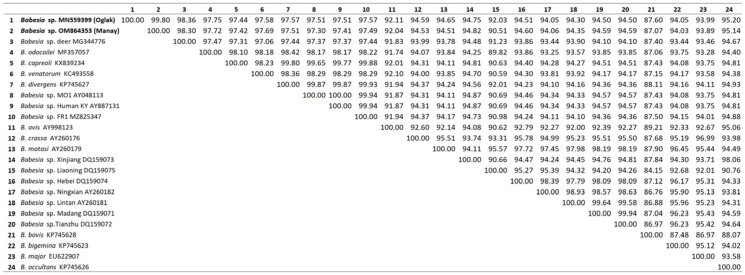
Pairwise distance matrix comparing the nearly full sequence of the *18S rRNA* genes of *Babesia* sp. (*manay*-OM864353, and *oglak*-MN559399) to other *Babesia* spp. Created by Clustal2.1. Data represent % identity (*p*-distance).

The nearly full-length sequence of the *18S rRNA* gene of new *Babesia* sp. was determined from DNA obtained from splenectomized goats (*manay* and *oglak*). Sequences were deposited in the EMBL/GenBank databases under accession number OM864353, and MN559399. These two sequences and previously reported sequences (KU714605- KU714606) are 99–100% similar to each other. The sequence identity of (%) between the newly recognized *Babesia* sequence and other targeted *Babesia* species is presented in Figure 3. Sequence comparison in BLAST showed that the *Babesia* sp. isolate identified in this study was different from all ovine *Babesia* species and genotypes currently available in the GenBank database. This isolate is 92.04–94.82% similar to the *B. ovis*, *B. motasi*, *B. crassa*, *Babesia* sp. Xinjiang, *Babesia* sp. Liaoning, *Babesia* sp. Hebei, *Babesia* sp. Ningxian, *Babesia* sp. Lintan, *Babesia* sp. Madang, and *Babesia* sp. Tianzhu species and genotypes that cause babesiosis in sheep and goats. The highest identity was observed with the *Babesia* species causing babesiosis in deer (*Babesia* sp. deer, *B. odocoilei*, *B. capreoli*) at 98.30–97.75%, and with the species and genotypes causing babesiosis in humans (*B. venatorum*, *B. divergens*, *Babesia* sp. MO1, *Babesia* sp. Human, *Babesia* sp. FR1) at 97.30–97.69% (Figure 3).

**Figure 4:**
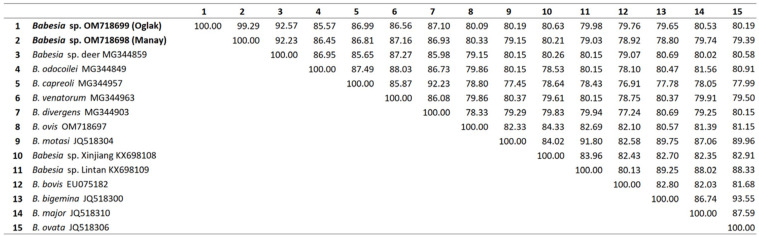
Pairwise distance matrix comparing ~900 bp of the *cox1* genes of *Babesia* sp. (*manay*-OM718698, and *oglak*-OM718699) to other *Babesia* spp. Created by Clustal2.1. Data represent % identity (p-distance).

The partial *cox1* sequence of new *Babesia* sp. obtained in this study was registered with GenBank under accession numbers OM718699 and OM718698. Nucleotide sequence identities showed that our *Babesia* sp. sequences were highly similar to *Babesia* sp. deer (MG344869, MG344859), with an identity of 92.23–92.57%. This isolate showed 85.57–86.45%, 86.56–87.16%, 86.56–87.16%, 86.93–87.10%, 80.09–80.33%, 79.15–80.19%, 80.21–80.63%, 79.03–79.98%, 78.92–79.76%, 78.80–79.65%, 79.74–80.53%, and 79.39–80.19% similarity to *B. odocoilei*, *B. capreoli*, *B. venatorum*, *B. divergens*, *B. ovis*, *B. motasi, Babesia* sp. Xinjiang, *Babesia* sp. Lintan, *B. bovis*, *B. bigemina*, *B. major*, and *B. ovata*, respectively (Figure 4).

**Figure 5:**
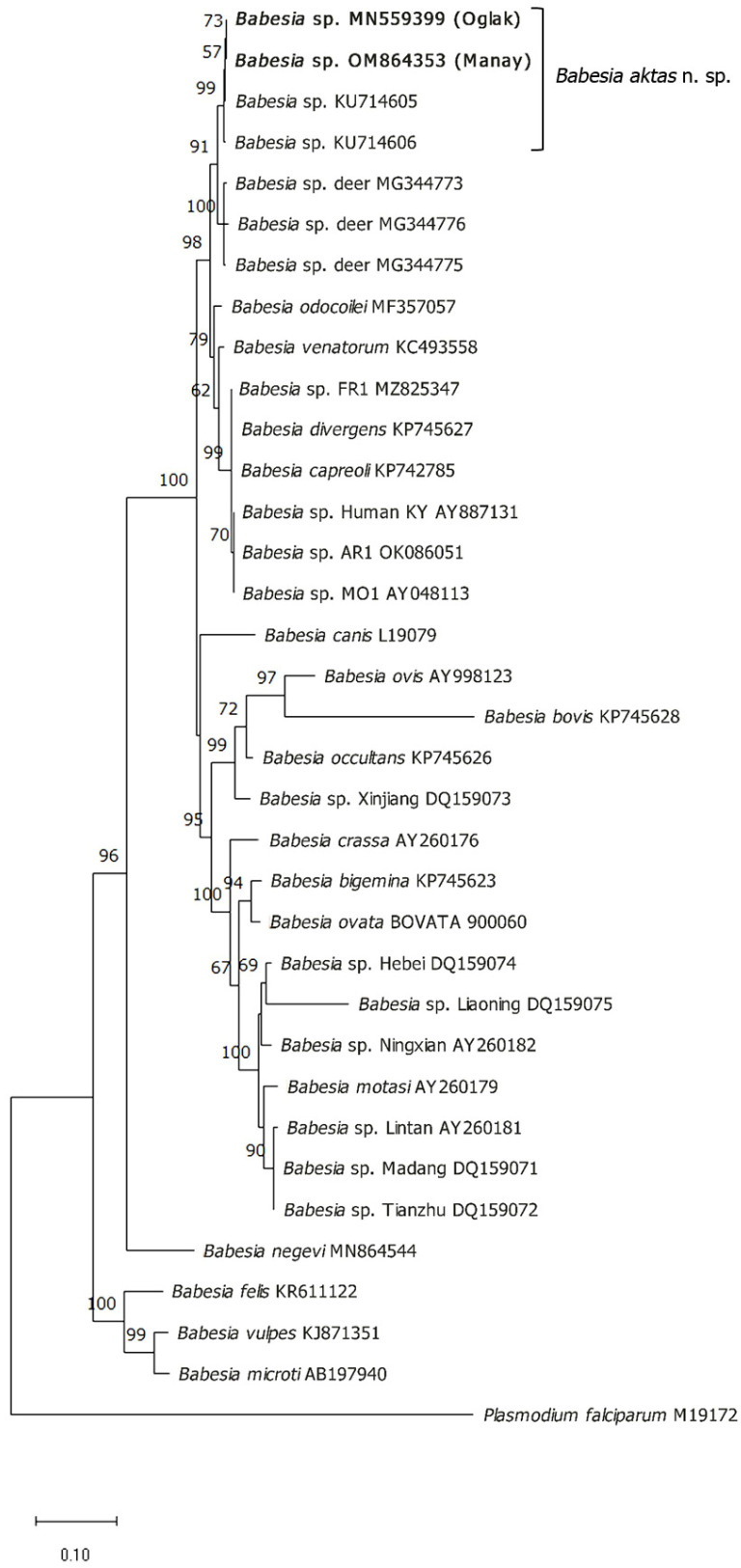
Phylogenetic analysis of *18S rRNA* sequences by maximum likelihood. The evolutionary history was inferred based on the Tamura-Nei (G+I) models. Each tree shows the phylogenetic relationship of new *Babesia* sp. determined in this study (bold letters) with other apicomplexan parasites. The percentage of replicate trees in which the associated taxa clustered together in the bootstrap test (1000 replicates) are shown next to the branches. Only bootstrap values > 50 are indicated next to branches. GenBank accession numbers are indicated on the right of each species name. *Plasmodium falciparum* (M19172) was used as an outgroup. The scale-bar represents the evolutionary distance in the units of the number of nucleotide substitutions per site.

**Figure 6:**
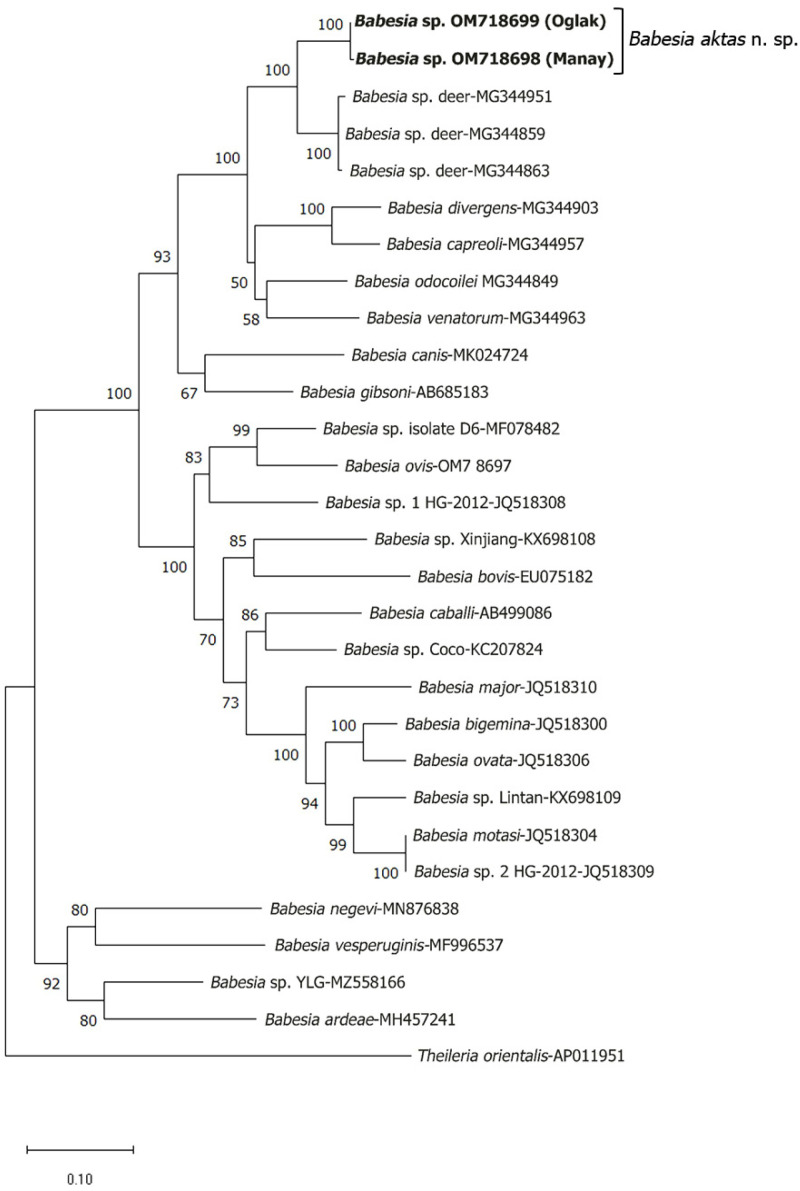
Phylogenetic analysis of *cox1* sequences by maximum likelihood. The evolutionary history was inferred based on the General Time Reversible model (G+I). Each tree shows the phylogenetic relationship of new *Babesia* sp. determined in this study (bold letters) with other apicomplexan parasites. The percentage of replicate trees in which the associated taxa clustered together in the bootstrap test (1000 replicates) are shown next to the branches. Only bootstrap values > 50 are indicated next to branches. GenBank accession numbers are indicated on the right of each species name. *Theileria orientalis* (AP011951) was used as an outgroup. The scale-bar represents the evolutionary distance in the units of the number of nucleotide substitutions per site.

A phylogenetic analysis was performed using *18S rRNA* and *cox1* sequences, including the *Babesia* sp. sequences identified in this study and other available *Babesia* sequences from the GenBank. Phylogenetic trees of *18S rRNA* and *cox1* sequences using the Tamura-Nei model (G+I) [29,31] and the General Time Reversible model (G+I) [29,32] are shown in Figure 5 and Figure 6, respectively.

## 4. Discussion

*Babesia* species are common in domestic and wild animals in tropical and subtropical regions around the world, including Türkiye, and cause clinical infections with high mortality rates [16,33,34]. Even though the *Babesia* parasite was discovered approximately 140 years ago, we still only know very little about it. More than 100 *Babesia* species have been described to date, and new species in vertebrates continue to be discovered in various parts of the world [2,3,4,9,10,11,12,13,14,28]. Molecular tests, which are mostly used for epidemiological studies in a given region, are widely used in research to detect parasites, confirm their presence, and discover new species or genotypes [35]. A molecular survey conducted in 2016 revealed the possibility of a new species in goats [16]. However, we were unable to determine whether this species actually infected goats or was present transiently in these animals. Recent review articles on this topic have stated that it is impossible to identify a new species based solely on gene sequences without observing the organism in question [35,36]. To demonstrate the presence of this parasite in goats, one animal (single infection) infected with new *Babesia* sp. was detected in the field study using the RLB method.

Experimental infections have been shown in splenectomized and dexamethasone immunodepression sheep and goats using the *Babesia* species. Because splenectomy reduces the host’s ability to control parasitemia, it allows for the large-scale expansion of previously undetected parasite populations to detectable and, in some cases, clinically significant levels [37,38]. High fever (42 °C), weakness, anorexia, anemia, and hemoglobinuria were observed in sheep with spleen-intact and splenectomized sheep during an experimental infection with *B. ovis* [39]. In another experiment with *Babesia* sp. Xinjiang, spleen-intact sheep did not develop parasitemia or clinical signs, whereas the splenectomized group developed fever (41.5 °C), parasitemia, and hemolytic anemia [28]. In a study conducted in China [24], blood from naturally infected sheep with *Babesia* sp. BQ1 (Ningxian) was inoculated into two splenectomized and spleen-intact sheep [24]. Body temperature increased (41.5 °C) in the splenectomized sheep on the fifth day after parasite inoculation, severe clinical findings with parasitemia developed, and the sheep died on the seventh day. On the fourth day after parasite inoculation, piroplasm forms were seen in the peripheral blood, and body temperature (42 °C) and parasitemia (1.9%) increased, and the sheep with severe clinical findings survived the disease [24]. Three splenectomized sheep were infected with *Babesia divergens*, the main cause of bovine and human babesiosis in Europe, using an in vitro stabilate, and all sheep developed a high fever and transient parasitemia between 6 and 9 days after infection [40]. The newly identified parasite *Theileria haneyi* was used in an experiment at the US-Mexico border. Blood from a horse infected with *T. haneyi* was administered to another spleenectomized horse in order to microscopically identify this novel parasite [14]. In this study, a splenectomy was used to morphologically characterize a new *Babesia* sp. identified by PCR and RLB. The first piroplasm was observed on the tenth day after splenectomy, and the parasitemia reached 1.9% on the 13th day. The parasitemia lasted 5 days in this goat, with no clinical signs other than high fever, anorexia, and death. Pure infected blood stabilate obtained in vivo from this animal was administered to another goat (*Oglak*) that was free of blood parasites (*Babesia*-*Theileria*-*Anaplasma*-*Mycoplasma* spp.). This animal was humanely euthanized after developing severe clinical signs (high fever, anemia), and necropsy revealed mild jaundice and anemia. According to Koch’s postulate, re-isolating this parasite from the second goat (Oglak) and demonstrating that it is identical to the original parasite is critical for identifying a new parasite [36].

*Babesia* parasites are classified into two groups based on their size: large (2.5–5.0 µm long) and small (1.0–2.5 µm long) [41]. The new *Babesia* sp. described in this study was included in the small group of *Babesia*. Although morphologically divided into two groups, large and small forms of the same parasite can be found. A sequence analysis of parasites thought to be *B. motasi* morphologically revealed that they were *B. ovis*, and that there were large and small forms of this parasite [42]. *Babesia crassa*, an isolate from Iran and member of the large *Babesia* group, differs from the others in that it has four parasites in one infected erythrocyte. *Babesia* sp. Xinjiang, a new type of *Babesia* isolated in sheep in China, is also included in the large *Babesia* group.

According to phylogenetic analyses, the *Babesia* sp. isolated in this study was genetically related to *Babesia* sp. deer, *B. odocoilei*, *B. venatorum*, *B. capreoli*, and *B. divergens*. There have been reports of *B. capreoli*, *B. venatorum*, and *B. divergens* in Europe [16,43,44]. *Babesia* sp. deer has been reported in the Czech Republic as an unnamed species in red (Cervus elaphus) and sika (*Cervus nippon*) deer [43]. Wapiti/elk, reindeer, and caribou are the natural reservoirs of *B. odocoilei*. [43,45]. The primary vector of *B. odocoilei* is *Ixodes scapularis* (Acari: Ixodidae), which causes fatal infections in cervids [45,46]. *Babesia odocoilei* has also been detected in *I. scapularis* ticks collected from domestic dogs and cats in Canada [47]. *Babesia* venatorum is capable of infecting humans, as well as chamois (*Rupicapra rupicapra*) and ibex (*Capra ibex*) in the Alpine region [48]. *Babesia venatorum* is known to infect humans in Europe, and the roe deer is its reservoir host. Human cases of *B. venatorum* have been reported in Europe and, more recently, China [49,50]. *Babesia venatorum* has been found in sheep populations, and it has been suggested that farm animals may play a role in the spread of this parasite [51]. *Babesia capreoli* has been found in a variety of deer species, with a particularly high prevalence in roe deer [44,52]. There have been no reports of infections in humans. *Babesia divergens* causes acute babesiosis in cattle and humans [53,54] and has been found in roe, red, and sika deer, chamois, and Alpine ibex (Capra ibex) in Europe [43,48,54]. During field studies, no natural clinical infections caused by new *Babesia* sp. were detected in domestic goats. In a phylogenetic analysis based on the *18S rRNA* gene region, *B. venatorum*, *B. odocoilei*, *B. capreoli*, and *B. divergens* were included in a clade proposed to be named as Babesids, and the host-specificity of this clade was reported to be lower than that of ungulibabesids (*B. bovis*, *B. bigemina*, *B. caballi*) [55]. The nucleotide sequence analysis revealed that *18S rRNA* and *cox1* sequences of our *Babesia* sp. were most similar to those of *Babesia* sp. deer, sharing 98.36% and 92.23% identity, respectively. In addition, phylogenetic analyses revealed that the new *Babesia* sp. and *Babesia* sp. deer formed a sister clade. Although useful for diagnosis, the *18S rRNA* cannot distinguish definitively between *Babesia* species and strains. For example, *B. divergens* (cattle-human-deer) and *B. capreoli* (roe deer), which are nearly identical (only three nucleotide differences) according to the *18S rRNA* sequence, are distinct species that infect different hosts. It has also been reported that the *Babesia* sp. FR1 isolate, which was identified in humans in France, differs from *B. capreoli* by only one nucleotide according to the *18S rRNA* sequence [56]. Consequently, the *cox1* gene locus has greater genetic diversity than the *18S rRNA* gene for determining phylogenetic relationships and distinguishing *Babesia* species [4,43,57]. The *cox1* sequences analysis revealed that the similarity between *B. divergens* and *B. capreoli* was 92.23%, as in the new *Babesia* sp. and *Babesia* sp. deer species. As a result, despite the fact that the *18S rRNA* gene regions of our newly discovered *Babesia* sp. and *Babesia* sp. deer were 98.30–98.36% similar, they were classified as two distinct species due to differences in their *cox1* sequences and for being identified in different hosts. A ~900 bp long mitochondrial *cox1* gene alignment of two *cox1* sequences from new *Babesia* sp. with their respective *cox1* nucleotide sequences from other piroplasmids confirmed that new *Babesia* sp. resulted in a strongly supported clade, confirming its identity as a new species.

## 5. Conclusions

In this study, the morphological and genetic characteristics of a new *Babesia* sp. previously described based solely on molecular analyses in goats were determined. There was a genetic relationship between this newly discovered *Babesia* sp. and other species reported in deer, mountain goats, cattle, and humans at various proportions. It is known that mountain goats inhabit the region where *Babesia* sp. was defined [17]. It is necessary to conduct additional research on the clinical status and vector competence of this parasite in goats (spleen intact/splenectomy) and other hosts (mountain goats, sheep, calves). Furthermore, the zoonotic significance of this parasite should be investigated, as it shares 97.5% similarity with zoonotic *B. venatoum*, *B. divergens*, *Babesia* sp. FR1, and *Babesia* sp. MO1 species and genotypes, as determined by the *18S rRNA* gene.

## 6. Taxonomic Summary

Family Babesiidae Poche, 1913

Genus *Babesia* Starcovici, 1893

*Babesia aktasi* n. sp. Ozubek & Aktas (Alveolata: Apicomplexa: Hematozoa: Piroplasmida).

Type-host: Domestic goat, *Capra hircus* Linnaeus, 1758

Type locality: Anamur (36°01′17″ N, 32°48′07″ E), Mersin, Turkiye.

Other localities: Unknown.

Description: Ring, paired pyriform, spectacle frame-like, and line forms were defined in erythrocytes. Except for the ring form, the cytoplasm was usually not prominent. The line form was very specific for this parasite.

Additional hosts: Unknown.

Vector: Unknown.

Location in host: *Babesia aktasi* n. sp. infects host erythrocytes.

Pathogenicity studies: not performed.

Material deposited: *Manay* and *Oglak* strains deposited as blood stabilate and genomic DNA at the department of Parasitology, Faculty of Veterinary Medicine Firat Univesity, Turkiye. GenBank accession numbers for type strain (OM718699 and OM718698; OM864353, and MN559399)

ZooBankLSID: urn:lsid:zoobank.org:act:2AB69D33-0FAA-40E6-8CC6-A54AE787CE80

Etymology: The species was named after Dr. Munir Aktas, who supervised the PhD thesis in which this organism was first identified.

## Figures and Tables

**Table 1:** The frequency (%) of tick-borne hemoparasites and hemotropic mycoplasmas (single and mixed infections) in goats detected by molecular tools (PCR and RLB) (*n* = 50).

No. Positive	Identified Pathogens				
	*Babesia* sp.	*B. ovis*	*T. ovis*	*A. ovis*	*Mycoplasma* spp.
2	+	−	−	−	−
1	−	−	+	−	−
3	+	+	−	−	−
4	+	−	+	−	+
3	+	−	+	+	+
1	−	+	+	+	+
14 (28%)	12 (24%)	4 (8%)	9 (18%)	4 (8%)	8 (16%)

## Data Availability

Data available in a publicly accessible repository.

## Supplementary Materials

The following supporting information can be downloaded at: https://www.mdpi.com/article/10.3390/pathogens12010113/s1, Table S1: Primers and probes used in the study. References [58,59,60,61,62,63] are cited in the supplementary materials.
