# Supplementary material for: Discovery of a Novel Species Infecting Goats: Morphological and Molecular Characterization of Babesia aktasi n. sp
Source: Pathogens. 2023 Jan 10;12(1):113. doi: 10.3390/pathogens12010113 (PMC9863244; doi:10.3390/pathogens12010113)
Supplement: Supplementary file 1 [file pathogens-12-00113-s001.zip › pathogens-2105722-supplementary.pdf]

Supplemenatary Table S1: Primers and probes used in the study.

Primers	Sequence (5’-3’)	References
Nbab-1F	AAGCCATGCATGTCTAAGTATAAGCTTTT	[9]
Nbab-1R	CTTCTCCTTCCTTTAAGTGATAAGGTTCAC	
RLB-F2	GACACAGGGAGGTAGTGACAAG	[20]
RLB-R2	biotin-CTAAGAATTTCACCTCTGACAGT	
Ec9	TACCTTGTTACGACTT	[18]
Ec12a	TGATCCTGGCTCAGAACGAACG	
16S8FE	GGAATTCAGAGTTGGATCMTGGYTCAG	[19]
B-GA1B	biotin-CGGGATCCCGAGTTTGCCGGGACTTCTTCT	
8F	AGAGTTTGATCCTGGCTCAG	[21]
1492R	GGTTACCTTGTTACGACTT	
F2	ACGAAAGTCTGATGGAGCAATA	[22]
R2	ACGCCCAATAAATCCGRATAAT	
BaFor1	ATAGGATTCTATATGAGTAT	[23]
BaRev1	ATAATCAGGTATTCTCCTTGG	
BaFor2	TCTCTTCATGGTTTAATTATGATAT	
BaRev2	TAGCTCCAATTGATAAAACAAAGTG	
**Probs**		
Catchall	AmMC6-TAATGGTTAATAGGA(AG)C(AG)GTTG	[58]
Theileria spp.	AmMC6-TGATGGGAATTTAAACC(CT)CTTCCA	[59]
T. ovis	AmMC6-TTTTGCTCCTTTACGAGTCTTTGC	[59]
T. separate	AmMC6- TCGGATGATACTTGTATTATC	[60]
T. lestoquardi	AmMC6-ATTGCTTGTGTCCCTCCG	[60]
T. uilenbergi	AmMC6-TGCATTTTCCGAGTGTTACT	[60]
T. luwenshuni	AmMC6-TCGGATGATACTTGTATTATC	[58]
Theileria sp. OT1	AmMC6-ATCTTCTTTTTGATGAGTTGGTGT	[59]
Theileria sp. OT3	AmMC6-ATTTTCTCTTTTTATATGAGTTTT	[59]
Theileria sp. MK	AmMC6-CATTGTTTCTTCTCATGTC	[61]
Babesia Catchall 1	AmMC6 -ATTAGAGTGTTTCAAGCAGAC	[62]
Babesia Catchall 2	AmMC6 -ACTAGAGTGTTTCAAACAGGC	[62]
B. ovis	AmMC6-GCGCGCGGCCTTTGCGTTTACT	[59]
B. motasi	AmMC6-ATTGGAGTATTGCGCTTGCTTTTT	[59]
B. crassa	AmMC6-TTATGGCCCGTTGGCTTAT	[59]
Babesia sp.	AmMC6-TGCCGTGAATCGACATTCGTC	[16]
Anaplasma/*Ehrlichia* catch-all	AmMC6-GGG GGA AAG ATT TAT CGC TA	[19]
A. phagocytophilum 1	AmMC6-TTGCTATAAAGAATAATTAGTGG	[63]
A. phagocytophilum 3	AmMC6-TTGCTATGAAGAATAATTAGTGG	[63]
A. phagocytophilum 5	AmMC6-TTGCTATAAAGAATAGTTAGTGG	[63]
A. phagocytophilum 7	AmMC6-TTGCTATAGAGAATAGTTAGTGG	[63]
A. phagocytophilum A-HGE	AmMC6-GCTATAAAGAATAGTTAGTGG	[63]
A. phagocytophilum A-D- HGE	AmMC6-GCTATGAAGAATAGTTAGTG	[63]
A. ovis	AmMC6-ACC GTA CGC GCA GCT TG	[19]

AmMC6: Amino group
